# State-of-the-art review and update of *in vivo* models of necrotizing enterocolitis

**DOI:** 10.3389/fped.2023.1161342

**Published:** 2023-04-04

**Authors:** Geoanna M. Bautista, Anjali J. Cera, Hala Chaaban, Steven J. McElroy

**Affiliations:** ^1^ Department of Pediatrics, Division of Neonatology, University of California, Davis, Sacramento, CA, United States; ^2^ Department of Pediatrics, University of Oklahoma Health Sciences Center, Oklahoma, OK, United States

**Keywords:** NEC = necrotizing enterocolitis, animal model, preclinical (*in vivo*) studies, intestinal injury, necrotizing/intestinal diseases/intestine

## Abstract

NEC remains one of the most common causes of mortality and morbidity in preterm infants. Animal models of necrotizing enterocolitis (NEC) have been crucial in improving our understanding of this devastating disease and identifying biochemical pathways with therapeutic potential. The pathogenesis of NEC remains incompletely understood, with no specific entity that unifies all infants that develop NEC. Therefore, investigators rely on animal models to manipulate variables and provide a means to test interventions, making them valuable tools to enhance our understanding and prevent and treat NEC. The advancements in molecular analytic tools, genetic manipulation, and imaging modalities and the emergence of scientific collaborations have given rise to unique perspectives and disease correlates, creating novel pathways of investigation. A critical review and understanding of the current phenotypic considerations of the highly relevant animal models of NEC are crucial to developing novel therapeutic and preventative strategies for NEC.

## Introduction

Necrotizing enterocolitis (NEC) remains a leading cause of morbidity and mortality in premature infants, with mortality rates as high as 10%–50% (1, 2). Clinically, NEC can rapidly progress from relatively mild feeding intolerance and abdominal distension to bowel ischemia and necrosis, fulminant septic shock, severe acidosis, multi-organ dysfunction, and death. Despite significant advances in neonatal clinical care in the last few decades, the prevalence of NEC has not significantly decreased globally (2, 3). Furthermore, the mechanisms driving the development of NEC remain poorly defined. This is in part because NEC is believed to result from a heterogeneous group of disorders or initiating pathways leading to a common final pathology (4). In addition, no current biomarkers predict the onset of NEC. Thus, it is difficult to study the mechanisms of NEC in human populations, making animal models that mimic NEC essential to determine the underlying pathophysiology and develop specific preventative and therapeutic targets (5).

Original models of NEC focused on adult animals undergoing experimental conditions such as ischemia-reperfusion injury, injections of pathogens into closed bowel loops, or combinations of hypoxia and hypovolemia (6). However, it quickly became apparent that the pathogenesis of NEC is a multifactorial process with four primary factors believed to be vital components driving disease manifestation. These include (1) immaturity of the intestine, (2) impaired mucosal barrier functions, (3) abnormal microbial colonization, and (4) dysregulated innate immunity (7). From this realization, the classic rodent model developed by Barlow et al. in 1974 became the mainstay of NEC research which involved exposing newborn rats to formula feeding, an oral inoculum of *Klebsiella pneumoniae,* and hypoxia (8). Since then, modifications have been made to the model, including adapting it to use in mice (9). In addition, new models have been developed that focus on the unique properties of the preterm infant, including the stage of intestinal development and immature immune systems (10). These have significantly contributed to our improved understanding of the mechanisms driving the increased susceptibility to intestinal injury in preterm infants and term infants with specific conditions associated with NEC.

Numerous animal models have been explored, including mice, rats, quails, rabbits, pigs, and baboons, each contributing to our understanding of NEC pathophysiology. However, given that NEC is a complex process with variable presentations and severity, no single animal model can truly and perfectly mimic NEC. Instead, each model captures a specific aspect of NEC, most aimed at recreating the predisposing clinical conditions that drive NEC susceptibility. In addition, animal models provide a means to manipulate variables that provide mechanistic insight and an ability to test therapeutic and preventative interventions in translatable preclinical models. This state-of-the-art review focuses on the highly relevant *in vivo* animal models of NEC, specifically the phenotypic considerations of each model and the research questions each model is best suited for. A comprehensive review of established animal models of NEC published since the 1960s was performed using search terms including but not limited to “necrotizing enterocolitis” “animal models”, “necrotizing enterocolitis murine/rat/piglet model,” “in vivo necrotizing enterocolitis,” “experimental necrotizing enterocolitis.” Once models were identified by keywords and previously published reviews, additional searches by corresponding authors and references were performed to identify the first publication using the original model and subsequent adaptations using a combination of Pubmed, Medline, and Google Scholar.

### Ethical, governance and regulatory considerations

An in-depth discussion on these issues is beyond the scope of this review and are well summarized elsewhere(11–13). However, it is important to highlight that statutory and regulatory frameworks have improved practice globally. More importantly there is paradigm shift towards a “culture of care” which we need to continue to nurture and disseminate.

### Risk factors for NEC

**Figure 1:**
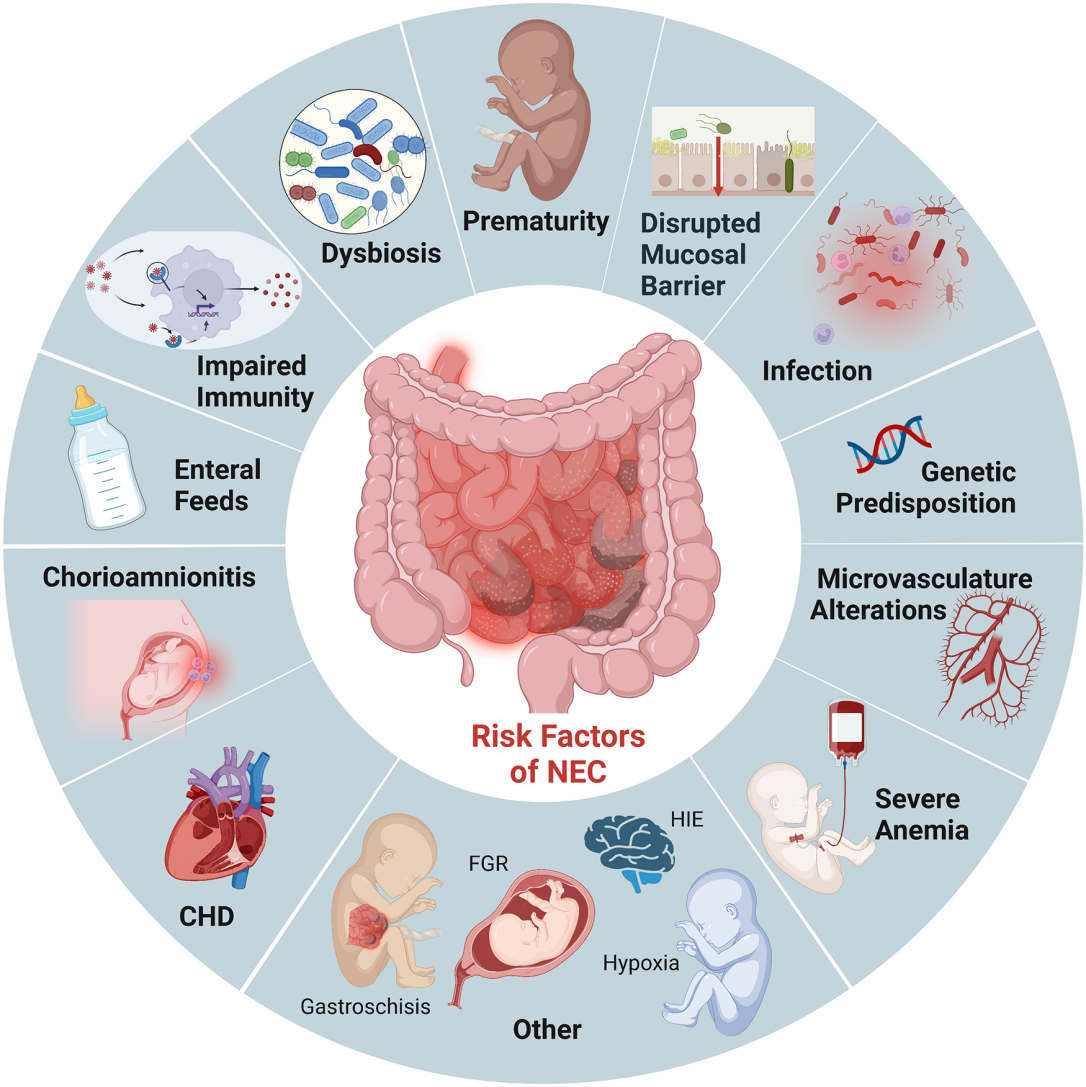
Established risk factors NEC. Illustration created with Biorender.com.

Prematurity remains the most critical risk factor for NEC. Roughly 90% of infants with NEC are born preterm, and the incidence is inversely related to gestational age (3). While the intestinal tract is one of the first organs to develop in humans, its development is not complete until term gestation. As a result, premature infants have immature intestinal barriers (impaired mucosal production, increased permeability), immunity (fewer Paneth cells, biochemically different mucous production, diminished regulatory T cells), and incomplete gut innervation with poor motility. Importantly, this combination of developmental immaturity of the preterm intestinal barrier function and the increased expression of toll-like receptor (TLR) 4 (14, 15) makes preterm infants particularly susceptible to the translocation of bacteria which can induce mucosal injury and lead to exaggeration of an already dysregulated inflammatory and immune response (Figure 1). These factors combine to induce further intestinal injury, ischemia, and necrosis seen in NEC (16).

In addition to prematurity, enteral feeding is a critical risk factor for developing NEC. Survival of the preterm infant depends on the delivery of adequate nutrition, often requiring supplementation with bovine and human-milk-based fortifiers for adequate growth. However, the combination of an immature intestine, a limited absorptive and digestive capacity, a dysbiotic microbiome, and delayed gut motility creates an intestinal environment marked by bacterial overgrowth and fermentation in the preterm infant (17, 18). These factors further contribute to the already dysbiotic and impaired mucosal barrier that renders preterm infants susceptible to mucosal injury (19). Studies have shown decreased incidence of NEC when infants are fed human milk (20). Furthermore, emerging evidence suggests that the absence of breastmilk and the critical components driving immunomodulation, barrier maturation, and growth promotion increase susceptibility to NEC rather than formula feeding itself (17, 21, 22). However, breastmilk does not completely prevent the development of NEC, and not all formula-fed premature infants develop NEC. We continue to lack complete mechanistic insight into how enteral feeding type can drive the increased susceptibility to intestinal injury, thus the critical need for multiple approaches and modeling to determine causality for intervention.

Other risk factors for developing NEC in the premature population include prolonged exposure to broad-spectrum antibiotics (23), severe anemia followed by transfusions (24), gastric acid suppression (25), sepsis/remote infection, and chorioamnionitis (26). While much of the recent studies have focused on the intestinal epithelium and inflammatory cells, given the histopathological characteristic of ischemia and necrosis seen in NEC, the microvasculature of the intestine is likely also involved. Establishing reduced nitric oxide synthase (eNOS) expression in patients with NEC has led to the recognition that decreased VEGF activity and expression in human neonates are independent risk factors for NEC (27). It is also important to note that NEC can also affect term neonates. However, NEC in this population typically occurs in conditions that compromise intestinal blood flow and oxygenation, such as ductal-dependent congenital heart defects (28, 29). Therefore, animal models that mimic ischemia/reperfusion injuries alone are likely more representative of this subset of neonates that develop NEC.

NEC has now been modeled in rats, mice, hamsters, piglets, rabbits, dogs, quails, and non-human primates, with piglets and rodents being the most commonly used. Perturbations of the intestinal environment in the neonate by directly or indirectly disrupting the protective mucosal epithelial barrier, innate immune functions, or the intestinal microvasculature/architecture are critical to inducing NEC-like phenotypes regardless of the animal model. It is essential to recognize that not all models of NEC have the same perturbations or disease phenotypes. Identifying predisposing factors and unique attributes for each model can help improve our understanding of NEC and is imperative for choosing the best model to answer the scientific question.

### Histopathology of NEC in humans and animal models

The most common diagnostic pathologic finding of NEC is pneumatosis intestinalis. This pathognomonic finding can be seen on radiograph imaging (x-ray and ultrasound), on gross examination of the bowel, and on histopathology. Pneumatosis represents intramural gas within the bowel wall produced by bacterial fermentation within the gut lumen. Other hallmark features in human NEC include portal venous gas, mucosal edema, epithelial sloughing/villous atrophy, secondary bacterial infiltration, vascular thrombosis, and discontinuous coagulative necrotic segments intestine or “skip lesions” that vary in depth of the affected intestine (29, 30).

While pneumatosis and other signs are utilized clinically, histological grading of NEC severity is the gold standard in rodent models. The original grading system described by Barlow et al. and subsequently validated by Caplan (31) and Dvorak (32) continues to serve as the basis for determining the incidence of and severity of NEC in rodent models today. In general, scoring is done on a Likert scale grading the extent of destruction of the intestinal mucosa: Grade 0—normal mucosa (intact epithelium); Grade 1—superficial epithelial sloughing or “lifting” (tip); Grade 2—mid-villous necrosis; Grade 3—complete villous necrosis; and Grade 4—complete loss of intestinal structure with transmural necrosis (31, 33). Generally, this follows one of two patterns depending on the model used: a top-down or bottom-up disease development (34). Additional features have been integrated, including separation of lamina propria, mucosal edema, coagulative necrosis, and depth of bacterial invasion. Scores of 2 or greater are considered to be representative NEC in humans.

The piglet model is unique in that the preterm piglet shares many overlapping features of gut anatomy, physiology, and microbiota with premature human infants (35). Thus, the grading system utilized in piglet models of NEC combines clinical features (e.g., abdominal distension, pneumatosis on imaging, cyanosis) with histological markers (coagulation necrosis, epithelial sloughing, and blunting mucosal edema, and leucocyte infiltration) to determine NEC-like intestinal injury (36). Furthermore, unlike most rodent models with NEC-like injuries occurring predominantly in the distal ileum, and taking 1–3 days to develop an injury, piglet models have an early onset of NEC (<24 h) that results in fulminant disease throughout the stomach to the large intestine, displaying a more widespread inflammatory response than typically seen in human neonates (37, 38).

### Modeling necrotizing enterocolitis *in vivo*: basic concepts

Given the limitations, expense, and difficulty of utilizing clinically obtained surgical specimens from neonates and human tissue-derived *in vitro* models (39), *in vivo* animal models have been crucial in elucidating the mechanisms contributing to the pathogenesis and severity of NEC (5). However, the wide spectrum of clinical manifestations and disease severity of NEC makes modeling NEC in animals particularly difficult, with no “perfect” model. Instead, most models developed to date are based on specific predisposing factors and the subsequent phenotypic effect on the mucosal epithelial barrier, microbiota/dysbiosis, and/or the hyperactivation of the innate immune system of the animal studied.

The earliest models of NEC were performed in adult animals that induced ischemic/reperfusion injuries by occluding the superior mesenteric artery (SMA) or surgically creating closed loops of small bowel (5). However, it was not until the 1970s that predisposing factors associated with NEC development in human neonates, including prematurity, formula feedings, and bacterial colonization, were incorporated into animal models (8, 26). The most widely used animal models of NEC to date are based on this original principle, integrating experimental conditions that increase the susceptibility to intestinal injury based on clinical factors associated with human NEC known at that time. This increased susceptibility is combined with an exposure to a triggering event that leads to intestinal dysbiosis, disrupted mucosal barrier, and an exaggerated inflammatory response triggering subsequent ischemia and necrosis characteristic of NEC. This multiple-hit methodology includes factors such as exposure to formula feeds, medications that cause mucosal injury or enhance microbial disruption, hypoxia ± cold stress, anemia, ischemia/reperfusion, or disruption or loss of critical regulators of the innate immune system such as Paneth cells.

### Specific animal models of NEC

#### Rat models of NEC

Barlow et al. (1974) described the first neonatal rat model of NEC, which demonstrated the importance of gut flora and lack of breastmilk (formula feeds) in the development of NEC-like injury (8), principles that are still pertinent today. This model was later expanded to include intermittent periods of hypoxia and hypothermia termed the HHF model, which serves as the foundation for many animal models of NEC subsequently developed (40). In addition, Caplan et al. (1994) later introduced bacterial pathogens in the formula given to neonatal rats, inducing manifestations of NEC-like intestinal injury, thus revealing a critical role of pathogenic bacterial colonization in developing NEC (31).

These original models have served as the basis for decades of subsequent models that have since modified, adapted, and improved these original concepts (41) (Table 1). However, there continues to be great variability in certain aspects of the rat models used today, including the use of both preterm and term neonatal rats, composition and frequency of formula feeds, duration, and degree of hypoxia and/or hypothermia. In general, rat pups are typically delivered *via* cesarean section or induction of labor by oxytocin administration, allowing for the avoidance of protective maternal milk feeds. The pups are then exposed to varying degrees and duration of hypoxia and/or hypothermia, followed by the introduction of a triggering agent such as lipopolysaccharide (LPS) and/or pathogenic bacteria (i.e., *Cronobacter sakazakii, Klebsiella*) administered enterally, intravenously, or intraperitoneally (52, 81, 82). These models generally take up to 3–5 days of exposure to various combinations of the above conditions before disease manifestation and development of NEC-like intestinal injury.

**Table 1:** Commonly used and relevant animal models of NEC.

Model (Abbreviation)	Animal (Age)	Protocol Time	Key Points	Ref.
Hypoxia Hypothermia Formula (HHF)	Rats (E20–21)	3–4 days	Commonly used model, basis of several current models across species. Adaptations typically include gavage feeds with hyperosmolar formula creating impaired barrier/dysbiosis and exposure to bacterial pathogen (either colonized or administered *via* orogastric tube) ± LPS following exposure to periods of hypothermia, hypoxia ( ± hyperoxia to stimulate clinical conditions in certain variations). Activates TLR-pathway.	(9, 32, 40, 42–45)
	Mouse (P0–10)	3–7 days	Widely used adaptation of rat HHF model with vast number of variations that often include exposure to bacterial pathogens (*Klebsiella, C. sakazakii, E. coli, “NECteria”)* ± LPS for increased disease manifestation. Commonly applied NEC induction protocol in transgenic mice testing specific genes/mechanisms (TLR4, VEGF, eNOS) ± exposure to various reagents/antibodies/modulators (amniotic fluid, HIF1α, IGF) to test therapeutic potential.	(9, 27, 33, 46–50)
	Piglet (Term, E115)	3–4 days	High rate of complications including 36% with pulmonary hemorrhage, 24% rectal perforations partly associated to technique (intubation, tonometer applied transanally), with severe manifestation of NEC.	(51)
Hypoxia Formula (HF)	Rats (E20–21, P0–3)	4–7 days	Requires exogenous or catheter colonization of bacteria to induce consistent NEC-like injury. Many variations utilized with varying exposures to hypoxia (decreased FiO2, 100% Nitrogen, etc) and different formula types (RMS, Ebsilac, human formula)	(8, 31, 52–56)
	Mouse (P0-P14)	3–4 days	Requires bacterial challenge ± LPS to trigger bacterial/inflammatory signaling; Important model for TLR4 signaling mechanisms.	(57, 58)
Formula Feeds (FF)	Mouse: SSC/Elecare (P8)	3 days	Use of hyperosmotic preterm human formula to induce NEC-like injury without exposure to hypoxia/hypothermia, has not yet been validated/replicated	(59)
	Mouse: Maltodextrin (P5–6 and P9–10)	10 days	Important model to study specific components (maltodextrin) of formula that create susceptibility to NEC. Consistent pattern of impairment with addition of maltodextrin combined Klebsiella (K) and/or hypoxia (H), worse with M/H. High survival rate after NEC induction protocol with milder severity.	(60)
	Piglet: Formula variations (E105–108)	1–4 days	Induction of NEC with formula feeding alone without exogenous hypoxic or hypothermic conditions. Prematurity of piglet (with transitional hypoxia and similarly impaired microvasculature to human premature infants), and presence bacterial colonization (no impact on germ-free piglets) necessary to induce intestinal injury.	(38, 48, 49, 61, 62)
	Piglet: Parenteral Nutrition (PN) (E105–109)	5 days	Gut dysfunction worsens with PN followed by FF, PN results in delayed gut maturation worsened by dysbiosis-induced FF. Several management changes in initiation of feeds, rate of feeding advancements, type of enteral feed, and PN-related applications derived from this model. Low true-NEC manifestation, wider distribution of disease to entire GI tract (including stomach, colon).	(51, 63–66)
Paneth Cell Disruption (PCD)	Mouse (P14–16)	16 h	Dithizone- or Diphtheria toxin-induced PC depletion. Model to study role of PCs in NEC, intestinal development closer to human development, model further simplified with either exposure to hyperosmotic formula (RMS) or bacterial challenge (*Klebsiella, NECteria*); short protocol time, TLR4-independent pathway	(10, 67–70)
Microvasculature Maldevelopment (MM)	Mouse: -VEGF/-IGF (E16–20, P0-1)	3 days	Models used to study intestinal vascular development and function using HHF NEC induction protocol in neonatal pups, using inhibition, deletion or down regulation on VEGF-related pathways. Fetal exposure to inflammation (using LPS) *in utero* (E16–20) followed by NEC induction at P0-1 also explored to determine chorioamnionitis impact on vasculature development and susceptibility to NEC. Addition of TNF shown to worsen NEC severity *via* decreased VEGF/VEGFR2 activity but prevented by DMOG (*via* HIF1a).	(27, 71, 72)
	Mouse: eNOS (P5)	4 days	Loss of eNOS in transgenic mice leads to greater gut and lung injury with altered inflammatory cascade, NO protective	(73)
Phlebotomy-induced anemia (PIA)	Mouse (P2)	10–12 days	Model investigating whether severe anemia ± RBC transfusion contributes to development and severity of NEC, activating TLR4-signalling mechanisms to drive inflammation and injury; Can be used to evaluate risk factor of iatrogenic anemia and gut perfusion.	(74, 75)
Antibiotic Exposure/Dysbiosis (ABT)	Mouse (P1–14)	14 days	Model to study role of prolonged antibiotic exposure leading to increased susceptibility to NEC when challenged at P14.	(76)
	Piglet (E 106)	5 days	Model to study effects of enteral vs. parenteral antibiotics in immediate post-natal period suggesting that enteral but not parenteral exposure protected from NEC, short duration (<5 days) of IV antibiotics with mild injury noted, no NEC.	(77)
Trinitrobenzene sulfonic acid (TNBS)	Mouse (P10)	1 day	Model using non-specific immunologic stimulant (TNBS) to induce NEC-like injury, highlighted critical role of gut microbiome with absence of injury in germ-free mice.	(78)
Dextran Sulfate Sodium (DSS)	Mouse (P3)	6 days	Adaptation of DSS model of IBD in adult mice, applied to neonatal mice to induce intestinal injury in the absence of hypoxia, hypothermia or LPS driven by humoral and cellular immune responses.	(79)
Anti-CD3 mAb	Mouse (P0)	2 days	Novel model illustrating the effect of T-cell inhibition to explore role of adaptive immunity in severity of NEC-like injury combined with dysbiosis from formula feeds (injury attenuated with antibiotics)	(80)

**Figure 2:**
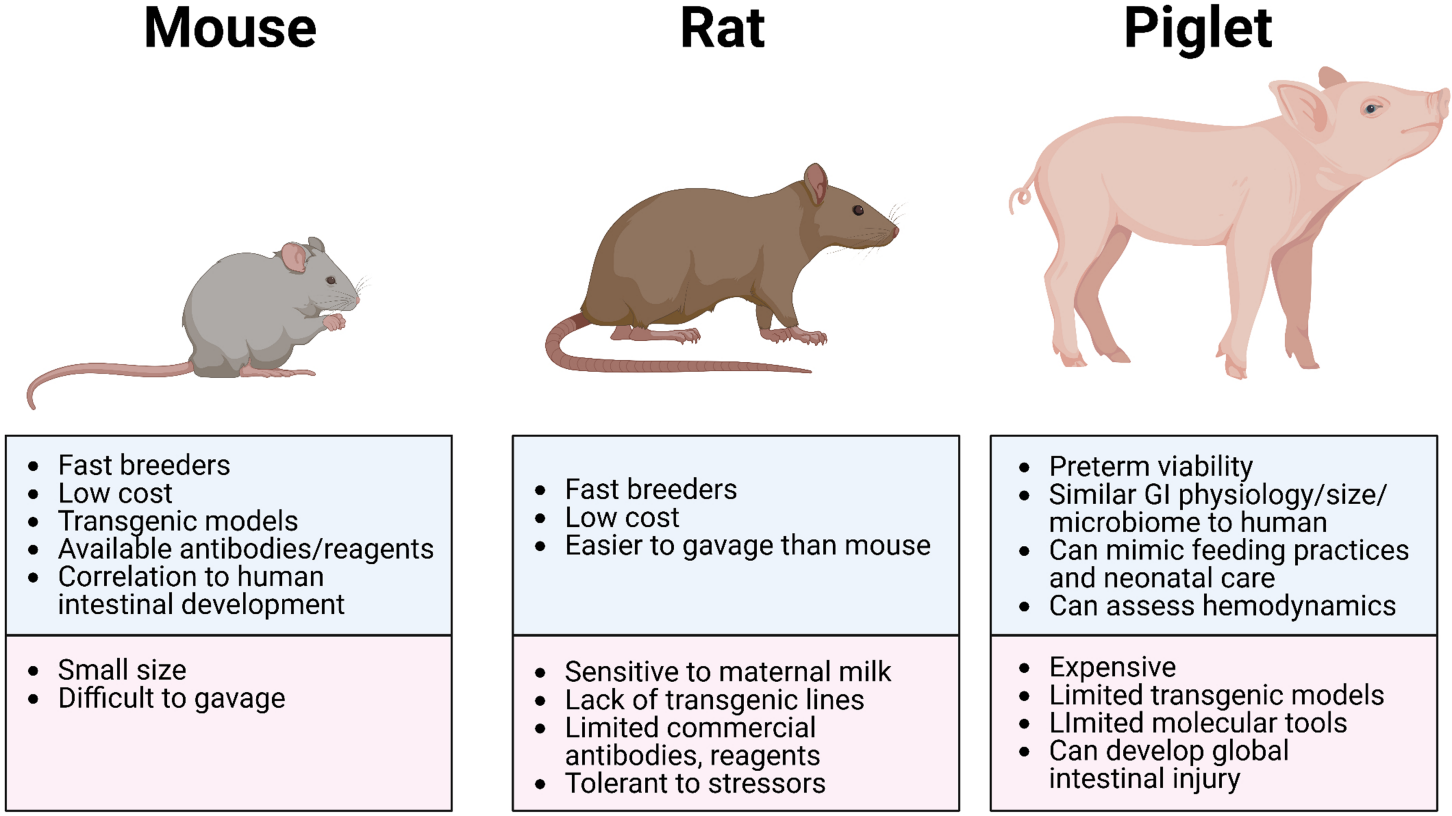
Most common animal models of NEC. Illustration created on Biorender.com.

The advantages of using rat models to study NEC include (i) the similarities in intestinal immaturity between premature human neonates and of neonatal rats, (ii) their preterm viability post-cesarean section, (iii) their resilience and relative tolerance of stressors used to induce NEC-like injury (which may also be disadvantageous due to the variable manifestation of disease), (iv) their reasonably larger size (compared to mice) making gavage feedings and other manipulations more feasible, and (v) their relative low cost with high reproduction rate (Figure 2 and Table 3). However, rat models have significant limitations in the ability to manipulate specific genes and pathways to aid in elucidating mechanistic processes and potential targets in disease development (83). Thus, studies use rat models primarily to test feasibility and safety of interventions such as probiotics, while mice models became more ideal for mechanistic studies and elucidating the roles of growth factors, stem cells, human milk oligosaccharides, and tumor necrosis factor (TNF) blockers (21, 84).

#### Mouse models of NEC

Many early and existing mouse models of NEC were an adaptation of the rat HHF model (Table 1). These models subjected mouse pups to some combination of formula feeds, hypoxia, hypothermia, LPS, and/or bacterial dysbiosis/colonization to induce NEC-like injuries (9, 82). More recently, Mihi et al. (2021) described a version of these adapted HHF models that removes hypothermia but includes hypoxic stress, formula supplemented with LPS, and enteric bacteria derived from an infant who died from NEC totalis, the most severe form of NEC (“NECteria”) (1). In addition, early mouse models of NEC initially attempted to deliver pups *via* cesarean section immediately before term to prevent exposure to maternal milk like in the rat models (9). However, subsequent studies confirmed that there is no need to immediately separate pups from their mothers since early dam feedings did not prevent the incidence of NEC (33). This is also demonstrated by the wide variability of postnatal ages of mice at the time of induction and subsequent disease manifestation of various mouse models of NEC.

Recognizing the emerging role of Paneth cells in the regulation of the innate immunity and protective mucosal barrier, the McElroy lab developed a two-hit model of NEC that requires both Paneth cell disruption and exposure to either enteral bacteria or formula feeds (68, 69). This model induces Paneth cell disruption by one of two validated methods: (i) chemically *via* the administration of dithizone, a heavy-metal chelator that reacts with zinc contained in Paneth cells leading to their disruption, and (ii) transgenically, using a human diphtheria-toxin receptor (DTR) that induce the selective necrosis of Paneth cells. This model does not require the combination of formula feeds, hypoxia/hypothermia, formula feeds, and bacterial challenge/dysbiosis to induce NEC, which most rodent models are based on. By limiting the number of experimental conditions and time required for disease manifestation (onset within 16 h vs. up to 5 days in other rodent models), this model may be more feasible. This model has uncovered new mechanisms and pathways that contribute towards the development of NEC that is independent of the well-studied TLR4 pathway and has now been validated and successfully replicated by other labs (76).

The advantages of using murine models of NEC include their relatively inexpensive cost, the ease of breeding, and the ability to genetically manipulate strains (Figure 2 and Table 3). In addition, mice are born relatively early with relatively immature intestines, which continue to develop postnatally. Based on the presence and abundance of 20 epithelial genes shared by mice and humans, the mouse intestinal epithelium has been shown to develop similarly to the human intestine from mouse birth (equivalent to a human fetus around 16–20 weeks) until the mouse reaches four weeks of age (equivalent to a term human infant), making the mouse an excellent model to study premature gut development (85). Furthermore, many of the biochemical and genetic pathways implicated in the development of NEC in mouse models have also been observed in clinical NEC, such as pathways involving TLR4, EGF, IgA, and HMGB1 (9, 86, 87). The primary disadvantage of using mice is their relatively small size, which makes them difficult to handle and gavage feed with formula, thus increasing the likelihood of complications and inconsistency. Still, mouse models of NEC have greatly advanced our understanding of the immature intestine and the factors contributing to injury susceptibility.

#### Piglet models of NEC

Touloukian et al*.* (88) were the first to describe a neonatal piglet model of NEC by inducing asphyxia followed by resuscitation, leading to hallmark features of intestinal necrosis. However, because this model utilized mature piglets (7–20 days old) and severe asphyxia approaches, Cohen et al*.* (51) modified this approach using moderate asphyxia (50% reduction in PaO2 ×30 min) in neonatal piglets (3–96 h old). Subsequent adaptations and modifications were made, shifting to the use of premature piglets without active asphyxia induction (89, 90).

With some minor variations, the piglet model of NEC generally involves the delivery of neonatal piglets at about 90% of full gestation (104–107 days of normal term at 114–118 days) (Table 1). Since the intestinal maturation of the piglet is not complete until a few weeks after birth, this period correlates with more premature intestinal physiology of human infants born at 75% of full gestation (28–30 weeks gestation) (91). Similar to the HHF rodent models, these piglets are exposed to a period of either natural or induced hypoxia/hypothermia followed by formula feeds to induce injury (91, 92). This model was later expanded to introduce the administration of total parenteral nutrition (TPN) prior to transitioning to enteral feeds. Exposure to TPN resulted in delayed intestinal growth and development that was characterized by mucosal atrophy, impaired mucosal barrier, and digestive functions that increased the development of NEC (65). Other piglet models of NEC include the combination of cow-based formula with high fat (3.5%) and ischemia/reperfusion (93) or *via* administration of iso-osmolar acidified casein solution into surgically created bowel loops in neonatal piglets (<3 days old and 2 weeks old) (94).

The greatest advantages of the piglet model are the size of the animal, similarities in metabolism and microbiome to humans, and a greater degree of similarities with human neonatal intestine, making this model highly translatable (Figure 2 and Table 3). Piglets can also be sustained prematurely and receive total parenteral nutrition (TPN) *via* central venous access, mimicking similar clinical situations and management as the preterm infant, making the piglet model truly unique (91). However, besides being extremely costly to maintain, piglets have limited molecular analytical tools, such as antibodies, and it is difficult to create transgenic strains for genetic manipulation. Additionally, while HHF induces similar histological changes that resemble NEC, the inflammation triggered in this model can be widespread involving the stomach and jejunum and not limited to the ileum as seen in human and rodent models of NEC. Regardless, given the similarities of clinical manifestations of NEC in piglets and human neonates, piglet models of NEC have been critical in elucidating specific aspects of the pathogenesis of NEC, evaluation of feeding regimen compositions and rates, preclinical drug studies for potential preventative and therapeutic targets, and the development of radiological diagnostic approaches (37, 95).

#### Other animal models

Other less frequently used animal models have been developed to study specific aspects of NEC, rabbit models of NEC consisted of variations of the HHF model with endotoxin, hypoxia, and cold stress (96), as well as intraluminal insults on closed intestinal loops (97, 98), resulting in the generation of free radicals and exaggerated release of leukotrienes causing NEC-like injury. In addition, a preterm rabbit model was also developed that incorporated anal blockage to simulate preterm neonates' poor intestinal function and dysmotility, resulting in NEC-like pathologic changes in the small and large bowel (99).

Notably, two studies described the development of spontaneous NEC in 5%–16% of preterm non-human primates (14, 100). In one study, baboons were delivered prematurely *via* cesarean section at 125 days gestation, correlating to 27 weeks gestation in humans (100). The baboons underwent identical management to premature neonates in neonatal intensive care units (NICUs) with mechanical ventilation, antibiotics, enteral feeds, etc., simulating the conditions that make them susceptible to NEC. Over two years, they reported the development of spontaneous NEC at the age of 7 to 18 days in 5% of the preterm baboons. In addition to the similar incidence and postnatal age, baboon NEC had a striking clinical, radiological, and histopathological resemblance to human NEC. The possibility of creating an NEC model in non-human primates would offer multiple advantages due to the high degree of genetic similarities, the similar gastrointestinal anatomy and physiology, and comparable immune response to humans. However, difficulty in animal procurement and lack of availability to many investigators, increased ethical considerations, and extremely high husbandry costs are major limitations for establishing such a model. Gnotobiotic quails have also been used to elucidate the mechanisms connecting specific bacterial species and the fermentation process of undigested nutrients that contribute to the development of NEC by inoculating germ-free quails (101, 102).

### Critical components and considerations when choosing a NEC model

#### Developmental stage correlation

As our understanding and management of infants with NEC evolved, so have the applicability of existing and new models (Table 1). Given that prematurity remains the most consistent risk factor for NEC, models have been developed to target the conditions of prematurity that may be driving the risk of NEC. Thus, understanding the stages of intestinal development in the model being used and how well correlated to the premature human infant will aid in determining whether the right model and age are being utilized.

The piglet model of NEC more closely matches the overall stage of development in the premature human infant (91). By delivering these animals at 90% of full gestation, there is better alignment with the premature state of human development on a multi-organ level, making the piglet model truly unique. The rat model is typically delivered just prior to term, closer to 94%–97% of full gestation, driven by inadequate lung development until that stage. Gut development, on the other hand, continues to mature postnatally, but unlike in the mouse model, many of the rat models of NEC rely on the prevention of maternal milk exposure to avoid its protective effects.

**Figure 3:**
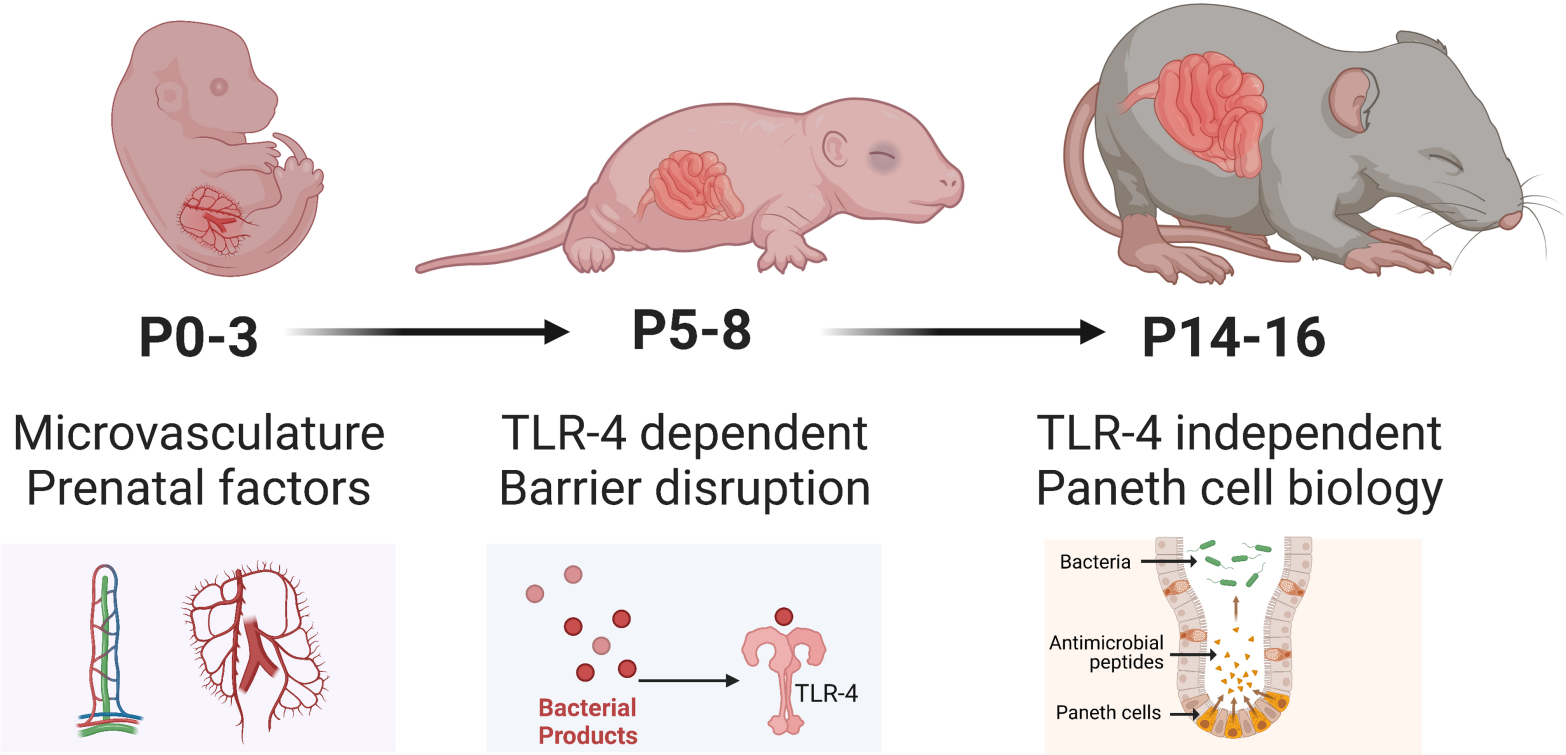
Developmental considerations of mice used to model NEC. Illustration created on Biorender.com.

While maternal milk is extremely protective in rat models, mouse models of NEC are still able to activate mechanisms that drive intestinal injury despite being dam fed, possibly due to a comparatively less developed intestinal epithelium. Compared to rat models, there is greater variability in the modeling of NEC in mice (Figure 3), particularly in the age of induction, ranging from postnatal day 0 (P0) to P16. This is particularly relevant since neonatal mice intestinal maturation continues postnatally, with the emergence of critical cell types and factors occurring at later time points. Since NEC most likely is a common endpoint of various pathways and pathogenetic mechanisms, disease manifestation at various postnatal ages is critical to determining which process may be triggered. For example, induction of NEC at earlier postnatal ages (P0-P7) in mice appears to trigger TLR4-related pathways despite the absence of Paneth cells in the neonatal mouse until at least P7. At the same time, NEC can occur with Paneth cell disruption in the absence of TLR4 (68).

#### Mucosal barrier disruption

The HHF model used in the rat, mouse, and piglet models of NEC is the foundation upon which subsequent models have developed (Table 1). This model applies a multiple-hit approach that disrupts the protective mucosal barrier and alters the microbiota environment creating more dysbiosis. This then leads to bacterial translocation and the triggering of the inflammatory cascade that follows in NEC.

The mucosal epithelium is the key interface between the environmental microbiota, the neonatal host system, and its immune system (76, 103). This physical barrier includes tight junctions which modulate permeability, goblet cells that produce mucus (aids the trapping of pathogens and absorption of nutrients), and Paneth cells (produces antimicrobial peptides and a critical regulator of the innate immune system and stem cell niche) (67, 69, 104). The mucosal barrier in premature infants is immature, with increased permeability or “leakiness” that can lead to altered gut microbiota, nutrient deficiencies, and bacterial translocation to systemic organs. Also, premature babies have decreased mucin production, impacting the ability to trap pathogens and allowing increased penetration of the epithelium (105). Several animal models of NEC mimic conditions that ultimately lead to the disruption of the mucosal barrier, subsequently triggering the inflammatory cascade characteristic of NEC.

#### Dysbiosis and prolonged antibiotic exposure

The intestinal microbiota is critical to maintaining epithelial barrier functions (106). The integrity of the mucosal barrier symbiotically interacts with the intestinal microbiota, protecting from the overgrowth of opportunistic bacterial invasion and promoting continued gut epithelium maturation. Changes in the healthy microbial populations are critical for postnatal intestinal development, particularly in the underdeveloped intestinal barrier of preterm infants (107–109). However, in the preterm infant, the intestinal microbiota is impacted by several often-unavoidable factors such as mode of delivery, antibiotic usage, type of enteral feeds, and need for blood transfusions (110), further increasing their susceptibility to developing NEC.

Numerous studies in mouse, rat, and piglet models of NEC have consistently demonstrated a link between bacterial colonization and the pathogenesis of NEC (111). In addition, several animal models have repeatedly shown a greater incidence of NEC-like lesions when animals are colonized or challenged with bacterial strains combined with an acute stressor to increase further susceptibility and disease manifestation. Other models that do not directly introduce a bacterial pathogen introduced variables that are now known to cause alterations in the microbiota populations, increasing the risk for bacterial translocation (31, 52, 68, 91).

Prolonged exposure to antibiotics, while often necessary in the premature population, has also been shown to increase the risk of developing NEC, likely due to the shifts in microbiota (112). Chaaban et al. (2022) describes a mouse model subjected to 10 days of the same empiric antibiotics used in neonates (ampicillin and gentamicin) of which more than half develop NEC following an oral bacterial challenge (76). This study nicely describes how prolonged use of systemic antibiotics lead to impairments in intestinal development, resulting in decreased cell proliferation, villi height, crypt depth, and numbers of goblet and Paneth cell expression. Interestingly, Birck et al*.* demonstrated that a shorter duration of enteral rather than parenteral antibiotics confers some protection from developing NEC in the preterm piglet model (77).

#### Enteral feeding types

While the exact etiology and pathogenesis of NEC remain poorly understood, enteral feeding type is recognized to play an important role (20, 113). It is surmised that enteral feeds combined with insufficient digestive capacities and an incompletely formed vascular system lead to bacterial overgrowth and increased metabolic demand on the immature intestine, further creating a susceptible environment to injury. Animal models typically utilize hyperosmolar formulas to aggravate the disruption of the mucosal barrier (Table 2). This concept has been used to mimic NEC in various animal models, particularly in rodent and piglet models. Importantly, hyperosmotic formula feeding is insufficient to create NEC-like injury, requiring a secondary insult such as hypoxia, cold stress, and/or bacterial pathogens to develop intestinal injury. Numerous studies have demonstrated that the lack of breastmilk and all the important components within it, rather than formula, increases susceptibility to NEC (69). Furthermore, animal studies have shown that the level of hyperosmolality to drive gut injury would need to be extremely high and beyond what is currently used in human neonates.

**Table 2:** Feeding type formulation and reported osmolarity/osmolality.

Feeding Type	Osmolality (mOsm/kg) Osmolarity (mOsm/l)	Models used	Ref.
Rat/mouse (dam) milk	352 mOsm/l	Mouse, rat	(114)
Rat milk substitute (RMS)	660–721 mOsm/kg	Mouse, rat	(70)
Hyperosmotic: 15 g Similac + 75 ml Esbilac	849 mOsm/kg	Mouse, rat	(22)
Diluted hyperosmotic: Similac lower iron + Esbilac	324 mOsm/kg	Mouse, rat	(22)
33% Esbilac	Not measured/reported	Mouse, rat	(47)
Elemental formula (Elecare)	455 mOsm/kg	Mouse	(59)
Similac Special Care (SSC)	303 mOsm/kg	Mouse	(59)
Elemental formula (Neocate)	360 mOsm/kg	Mouse	(57)
Elemental formula (Pregestimil)	710 mOsm/kg	Dog	(115)
Term formula (Similac)	295 mOsm/kg	Mouse, dog	(115)
Preterm formula (Neosure)	298 mOsm/kg	Mouse	(59)
Pig milk (colostrum, preterm)	344 mOsm/l	Piglet	(38)
Pig milk (unfortified, donor)	312 mOsm/kg	Piglet	(116)
Commercial pig milk formula	481 ± 41 mOsm/kg	Piglet	(117)
Custom pig milk formula	182 mOsm/l	Piglet	(38)
Hyperosmotic milk formula + sorbitol	872 ± 32 mOsm/kg	Piglet	(117)

The models that utilize formula as an inciting factor to develop NEC-like injury utilize additional aspects of prematurity in combination or with an added inflammatory response. Formula-feeding-associated dysbiosis, in combination with factors that increase mucosal inflammation, has been shown in several models. As a recent example, Singh et al. (2020) describe a model that uses a maltodextrin-dominant formula, combined with either hypoxia and/or bacterial challenge with *Klebsiella* induce NEC in P5–6 and P9–10 murine pups without hypothermia (60).

#### Importance of innate immunity in modeling NEC

Premature neonates have intestinal immaturity that leads to a disrupted mucosal barrier, an underdeveloped immune defense system, altered vascular development and tone, and delayed enteric innervation (110). Intestinal inflammation and sepsis can develop when exposed to luminal bacteria that is impacted by enteric feeds, antibiotic exposure, and delivery method. The neonatal intestine must quickly respond to the presence of both “good” and “harmful” bacteria after birth, making the role of the innate immune system and mucosal barrier critical to avoiding injury. Animal models have been vital to characterizing the massive inflammation that occurs with NEC that appears to be triggered by either a TLR4-driven pathway or a TLR4-independent mechanism *via* Paneth cell disruption.

The most widely studied mechanism contributing to NEC pathogenesis is the role of Toll-like receptor 4 (TLR4), a receptor for LPS, a component of the outer membrane of Gram-negative bacteria critical for developing NEC (14). A large body of work Hackam et al*.* and others has shown that the activation of TLR4 results in the inappropriate activation of the NF-kB pathway, resulting in mucosal damage *via* the production of proinflammatory cytokines, leading to damage of the intestinal mucosa. This then leads to bacterial translocation, further activating endothelial TLR4 leading to a reduced expression of the nitric oxide-generating enzyme eNOS in mice and further activating the inflammatory cascade in NEC (118, 119). In addition, TLR4 activation can also significantly inhibit the β-catenin signaling that is important for enterocyte proliferation in the ileum of newborn mice, which further leads to apoptosis and can lead to NEC (120).

Genetic alterations in the TLR4 pathway have also been found to increase susceptibility to NEC in humans. This includes variants of single immunoglobulin interleukin-1-related receptor (SIGIRR), which is associated with the inhibition and regulation of TLR signaling. Variants of SIGIRR have been associated with widespread inflammation and severity in NEC (15, 121). This was confirmed in SIGIRR −/− transgenic mice subjected to experimental NEC, leading to increased intestinal inflammation, apoptosis, and NEC severity (122).

However, TLR4 activation is not always associated with the development of NEC in premature infants, and NEC can develop in the absence of Gram-negative bacteria (123). An alternative mechanism was further established in the murine model of Paneth cell disruption that demonstrated that NEC-like intestinal injury could occur in TLR4 −/− mice subjected to Paneth cell disruption developed by the McElroy et al. (68). Human neonates with NEC have decreased expression of Paneth cells (124). Paneth cells are critical regulators of the innate immunity of the gut, producing essential antimicrobial peptides in the epithelium as part of the mucosal epithelial barrier and regulating the innate immune system (104). The innate immune system of the gut requires a careful balance between maintaining homeostasis on the one hand and rapid inflammatory response to pathogens and other threats on the other; thus, impaired Paneth cell function can create a proinflammatory state more susceptible to injury.

#### Modeling impaired microvasculature in NEC

One of the hallmark features of NEC is intestinal ischemia and necrosis. Earlier models attempted to recapitulate the ischemia that is believed to contribute towards the development of NEC. These models typically involved the occlusion of the superior mesenteric artery (SMA), effectively blocking blood flow to the small bowel and then allowing for reperfusion. However, these models replicated ischemia that occurs before NEC without also inducing inflammation, thus not an accurate model of NEC (28, 97, 125). Although not directly targeted, several models developed and currently utilized have some component that drives the ischemic changes seen in NEC. Whether it is hypoxia exposed *via* subjecting the animal to decreased oxygen concentration or nitrogen gas or “transitional” hypoxia that occurs when animals such as the piglet are delivered prematurely and require some mode of oxygen support.

Neonates, particularly premature infants, are uniquely vulnerable to hypovolemic or ischemic injury to the intestine compared to adults in part due to their relatively low resistance to blood flow (126). Postnatal hypoxia and other diseases that result in decreased blood flow, disruption of intestinal vascular development, and /or oxygen delivery resulting in impaired perfusion increase the risk of NEC in neonates and experimental animal models (42, 127, 128). Preterm infants with NEC also have been shown to have increased levels of TLR4 with reduced nitric oxide synthase (eNOS) expression, suggesting that intestinal endothelial dysfunction by endothelial TLR4 activation contributes to the development of NEC (118, 129). The role of inflammation is thus believed to trigger a secondary vasoconstriction that worsens the intestinal ischemia process leading to a vicious cycle of ischemia and inflammation characteristic of NEC (130).

Work done by De Plaen and colleagues have advanced our understanding of the mucosal microvasculature that is impaired in NEC, explicitly highlighting the importance of VEGF and VEGF-receptor 2 signaling pathways (27, 71). Specifically, this group has shown that inhibition of VEGFR2 with kinase inhibitors led to more severe intestinal necrosis with a higher mortality rate, decreased endothelial cell proliferation, and decreased microvascular network density. While the administration of macrophage-derived IGF-1, which promotes VEGF expression and endothelial cell proliferation, leads to protection against experimental NEC. These models applied a modified HHF NEC induction protocol on neonatal P0 transgenic mice. Data gathered from these experimental models are critical to our understanding of how the most commonly utilized models of NEC can result in ischemic changes coupled with a dysregulated inflammatory response (either *via* bacterial/LPS exposure or PC disruption), making this a truly unique aspect of studying the pathogenesis of NEC (71, 72).

#### Anemia and packed red blood cell (pRBC) transfusions in the development of NEC

Premature infants often develop severe anemia either early on secondary to iatrogenic blood loss from lab draws/procedures or later classically as anemia of prematurity, which is related to several factors, including insufficient erythropoietin production, immature bone marrow functions, high turnover of neonatal RBCs with shorter half-lives, infections, and nutritional deficiencies (74, 131). In addition, anemia alone has been shown to directly alter the intestinal barrier (increased mucosal hypoxia and barrier permeability) and innate immunity (increased proinflammatory macrophage activity) in a neonatal mouse model of phlebotomy-induced anemia (PIA) (75).

Mohankuma et al*.* (2019) combined the PIA model with RBC transfusions, creating a novel model to determine the combined and separate effects of each (74). In this study, severe anemia was found to cause inflammatory changes in the intestinal mucosa with macrophage infiltration, and the subsequent RBC transfusions further activated these cells *via* a TLR4-mediated mechanism to cause injury. Transfusion in anemic but not control mice was associated with intestinal injury within 28 h after transfusion, characterized by coagulative necrosis, inflammation, submucosal edema/separation, and interstitial hemorrhages (74). These studies highlight how severe anemia is an independent risk factor for NEC and that transfusion-associated NEC occurs only in the setting of severe anemia, likely due to a similar phenomenon as seen in ischemia/reperfusion models of NEC.

#### Other inflammation and immune-modulating approaches to NEC

Other models have been developed that attempt to induce the exaggerated inflammation seen in NEC. For example, Mohankuma et al*.* (2017) described a model that incorporates the enteral administration of trinitrobenzene sulfonate (TNBS), a non-specific immunologic stimulant that leads to an increase in chemotaxis for macrophage infiltration, resulting in a mucosal injury similar to that of NEC. In this model, TNBS was administered *via* gavage and enema to 10-day-old pups to induce enterocolitis. Interestingly, this model is ineffective when applied to germ-free mice, illustrating the critical role of the gut microbiota in developing TNBS-induced enterocolitis and NEC-like injury (78, 132).

Ginzel et al. (2017) administered formula containing dextran sodium sulfate (DSS), a mucosal irritant, to 3-day-old pups, which resulted in NEC-like disease of the small and large bowel in the absence of hypoxia or hypothermia (79). This model resulted in NEC-like lesions with both humoral and cellular immune responses throughout the intestine. This model is unique in that mucosal tissue damage was induced in the absence of any physical stressors in a relatively short period and produced a greater degree of intestinal injury than LPS alone.

Klinke et al. (2020) developed a mouse model that targeted the inflammatory cascade that occurs in NEC by altering neutrophil concentrations. In this model, neutrophilia by the administration of G-CSF leads to an increase in the disease manifestation of NEC when induced using hypoxia, formula, and LPS (133).

Subramanian et al. (2022) recently described a model of NEC that combines formula-feeding-associated dysbiosis with mucosal inflammation driven by anti-CD3 mAb treatment. This model uniquely illustrates the potential role of T-cell inhibition using anti-CD3 mAb. In addition, the severity of the NEC-like injury was attenuated with the administration of antibiotics and dam feeds (80).

## Conclusion

The multifactorial processes driving disease manifestation in NEC makes the development of an exact animal model of NEC difficult, if not impossible, to achieve. Instead, each unique model provides a different perspective on how multiple factors independently lead to the alteration of complimentary and overlapping signaling pathways that ultimately lead to NEC-like injury (Table 3). While Barlow's original neonatal rat HHF model continues to be the foundation on which many of the current models are based, unique approaches and considerations have emerged that offer new insight into the predisposing factors, pathogenesis, and more global effects of NEC. In addition, the continued advancement of molecular tools, data and collaborative science allows the discovery of new aspects and correlates to the human conditions of NEC that we seek to answer. Best practice in science requires the use of animal models only when other alternatives are not applicable, but because of the multifactorial pathophysiology of NEC and the difficulty obtaining human samples, animal models are needed to move the field forward (134). In developing these models, one must make every effort to implement the “3Rs” to guide the humane treatment of animals used in research. These include ***r***educing the number of animals used in research, ***r***efining procedures and studies to minimize pain, and ***r***eplacing animal experiments with *in vitro* models whenever possible (135). The Animal Welfare Act and specific governing bodies such as the Institutional Animal Care and Use Committee (IACUC) in the U.S. have been established to specifically aid research institutions and investigators in maintaining ethical practices and the most efficient use of animals in all research endeavors (136, 137).

**Table 3:** Animal models of NEC- advantages and limitations.

	Mice	Rat	Pig
Advantages	High reproductive rate Genetically modifiable Commercially available tools (existing antibodies, primers) Postnatal intestinal development Ability to induce NEC at various ages	High reproductive rate Relatively larger size than mouse Easier to gavage feed than mice Neonatal rats more resilient than mice	Preterm viability Ability to evaluate perfusion/hemodynamics Can perform sequential lab work Ability to mimic identical feeding practices (formula, TPN) and clinical exposure Similar GI physiology/size to human neonates
Limitations	Difficult to gavage feed Require regular feeds for hydration and glucose regulation	Lack of transgenic lines High endotoxin/bacterial tolerance Requires c-section to avoid dam milk	Limited molecular diagnostic tools Can develop global intestinal injury
Cost	Low	Low	High
Ideal for:	Elucidating mechanisms, pathways and single gene effects driving pathogenesis	Testing safety/feasibility Temporal biomarker studies	Translational evaluation for therapeutic strategies
Models:	HF, HHF, ABT, PCD, PIA, I/R, MHK, FF	HF, HHF, I/R	HHF, ABT, I/R, FF, FF/PN

HHF, hypoxia-hypothermia-formula feeding; ABT, antibiotic exposure; PCD, Paneth cell disruption; PIA, phlebotomy-induced anemia; I/R, Ischemia/reperfusion; MHK, Maltodextrin ± hypoxia ± Klebsiella; FF, formula feeding; PN + FF, parenteral nutrition followed by formula.

By understanding the basis of each model that currently exists and the unique aspects it can provide, new and current investigators will be able to determine the best tools available to elucidate the particular aspect of NEC they seek to explore further. By directing our efforts and using the optimal model, we can further delineate the various pathways disrupted in NEC, determine how modifiable factors such as enteral feeding types and environmental exposures specifically impact these pathways, and uncover potential genetic susceptibilities, leading to the successful identification of novel therapeutic targets and prevention strategies that will be crucial to our vision of a world without NEC.
